# Serial Observations and Mutational Analysis of an Adoptee with Family History of Hypertrophic Cardiomyopathy

**DOI:** 10.4061/2010/697269

**Published:** 2010-03-11

**Authors:** Bronwyn Harris, Jean P. Pfotenhauer, Cheri A. Silverstein, Larry W. Markham, Kim Schafer, Vernat J. Exil, Charles C. Hong

**Affiliations:** ^1^Center for Inherited Heart Disease, Vanderbilt University School of Medicine, Nashville, TN 37232, USA; ^2^Division of Medical Genetics and Genomic Medicine, Vanderbilt University School of Medicine, Nashville, TN 37232, USA; ^3^Division of Cardiovascular Medicine, Vanderbilt University School of Medicine, Nashville, TN 37232, USA; ^4^Vanderbilt Heart and Vascular Institute, Vanderbilt University School of Medicine, Nashville, TN 37232, USA; ^5^Division of Pediatric Cardiology, Vanderbilt University School of Medicine, Nashville, TN 37232, USA; ^6^Research Medicine, Veterans Administration TVHS, Nashville, TN 37212, USA

## Abstract

Hypertrophic cardiomyopathy (HCM) is an inherited cardiac disease with an autosomal dominant mode of transmission. Comprehensive genetic screening of several genes frequently found mutated in HCM is recommended for first-degree relatives of HCM patients. Genetic testing provides the means to identify those at risk of developing HCM and to institute measures to prevent sudden cardiac death (SCD). Here, we present an adoptee whose natural mother and maternal relatives were known be afflicted with HCM and SCD. The proband was followed closely from age 6 to 17 years, revealing a natural history of the progression of clinical findings associated with HCM. Genetic testing of the proband and her natural mother, who is affected by HCM, revealed that they were heterozygous for both the R719Q and T1513S variants in the cardiac beta-myosin heavy chain (*MYH7*) gene. The proband's ominous family history indicates that the combination of the R719Q and T1513S variants *in cis* may be a “malignant” variant that imparts a poor prognosis in terms of the disease progression and SCD risk.

## 1. Introduction

Hypertrophic cardiomyopathy (HCM), characterized by thickened ventricles, cardiomyocyte hypertrophy, and myofibrillar disarray, is a relatively common inherited cardiac condition, affecting approximately 1 in 500 individuals. HCM, which is the leading cause of sudden cardiac death (SCD) in youth, causes significant morbidity and mortality in affected individuals [1]. As a number of mutations in the sarcomere genes have been causally linked to HCM, genetic testing of first-degree relatives of HCM patients is recommended to identify those at increased risk for developing HCM. One of the most frequent HCM susceptibility genes is the cardiac beta-myosin heavy chain gene (*MYH7*), which is mutated in approximately 15% to 30% of all HCM [1]. Specific mutations in *MYH7* have also been found to be associated with severe disease, including greater hypertrophy, younger age at diagnosis, and worse prognosis [1, 2].

## 2. Case Report

**Figure 1:**
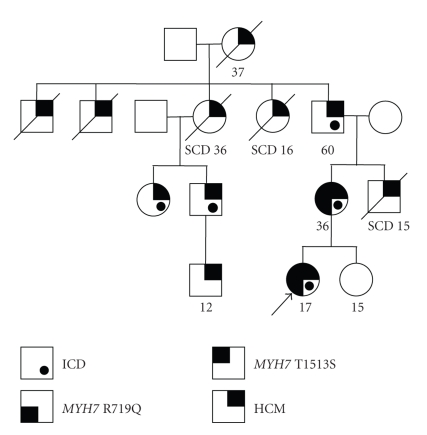
Pedigree of the adoptee. The proband is highlighted by the arrow. The proband carries two sequence variations within the *MYH7* (cardiac beta-myosin heavy chain gene): 2156G>A (R719Q) change in exon 19 (marked by a shaded area on upper left hand corner), and 4537A>T (T1513S) on exon 33 (the shaded area on the lower left hand corner). Cardiac hypertrophy is marked by shaded area on upper right-hand corner). SCD: sudden cardiac death. ICD: internal cardiac defibrillator placed (dot).

The proband, an adopted six-year-old girl, and her four-year-old sister were brought to a pediatric cardiologist for evaluation, largely at the insistence of the natural maternal grandmother (MGM), who had maintained contact with the adoptive parents. At the time of the initial evaluation, the proband's family history, obtained through the natural MGM, was notable for hypertrophic cardiomyopathy (HCM) in her maternal uncle (diagnosed on autopsy following SCD at age 15), maternal grandfather (s/p ICD for HCM), and two maternal great aunts (SCD at age 16 and 36) (Figure 1). At the time of the maternal uncle's death, the cardiac status of the proband's mother was deemed “normal.” On initial evaluation, the proband was asymptomatic with a normal physical exam, a normal electrocardiogram (ECG) and a normal echocardiogram. Given their significant family history for HCM, the proband and her sister were followed at 1-2 year intervals.

At 8, 11, and 13 years, the proband was largely asymptomatic. The electrocardiograms showed no evidence of ST changes, left ventricular hypertrophy (LVH), or T-wave changes. The echocardiograms were completely within normal limits. She remained a very active child playing soccer and basketball without any exercise intolerance. At 13 years, the proband experienced a syncopal episode lasting a few minutes that was attributed to a vasovagal event. Her ECG and Holter monitoring at the time did not show any abnormalities. 

**Figure 2:**
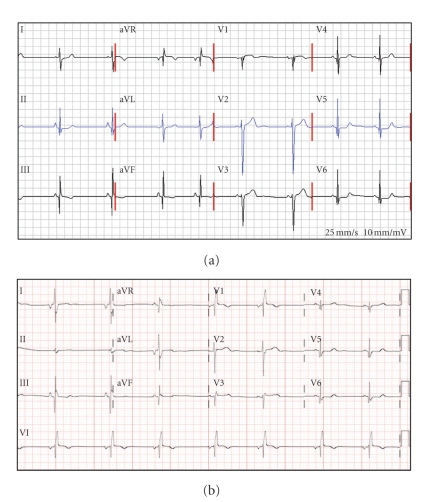
Evolution of ECG in the proband. (a) The proband's ECG at age 14 years. It is notable for Q-waves in lateral and inferior leads with nonspecific ST- and T-wave changes. (b) The proband's ECG at age 15 years. It is notable for right bundle branch block with left posterior fascicular block, Q waves in lateral, and inferior leads.

At 14 years, the proband continued to be athletic and denied any symptoms. Her physical exam and echocardiogram were normal. However, the proband's ECG did show nonspecific ST-T wave changes (Figure 2(a)). At this time, genetic testing was discussed with her and her adopted parents, but the family opted to defer testing.

At 15 years, the proband's ECG showed right bundle branch block, right posterior fascicular block, and Q waves in the lateral and inferior leads (Figure 2(b)). Subsequent cardiac evaluations, including an exercise stress test, echo, and MRI, however, were normal, without any signs of myocardial ischemia or hypertrophy. The proband denied any symptoms or exercise intolerance. 

**Figure 3:**
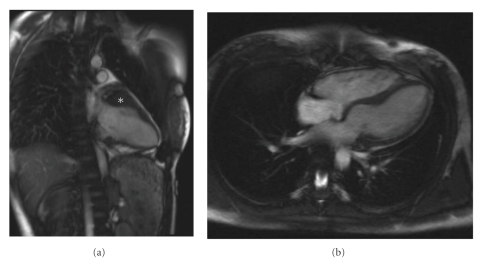
Focal hypertrophy noted on the proband's cardiac MRI. (a) 2-chamber end-diastole view. *marks the focal thickening (2.7 cm, thick × 1.8 cm, length) of the anterior wall at the base of the left ventricle. (b) 4-chamber end-diastole view.

At 17 years, the proband's echocardiogram showed very mild concentric LVH (thickness 1.1 cm, Z-Score of 2), with evidence of diastolic dysfunction (maximal E : E' ratio=12). An exercise stress test did not reveal any evidence of ischemia or ectopy, and Holter monitoring was unremarkable. At this time, the proband was restricted from participating in competitive athletics, and genetic testing was performed (see below). An MRI performed two weeks later showed normal overall left ventricular mass at 67 g/m sq and normal interventricular septal and posterior wall thickness, but also revealed a prominent 2.7 cm thickening of the anterior wall at the base of the left ventricle (Figure 3). 

The genetic testing, performed by the Harvard-Partners Laboratory for Molecule Medicine (http://www.hpcgg.org/LMM/), involved sequencing the coding regions and splice sites of the genes encoding the sarcomeric proteins cardiac alpha-actin (*ACTC*), cardiac myosin-binding protein C (*MYBPC3*), cardiac beta-myosin heavy chain (*MYH7*), myosin regulatory light chain (*MYL2*), cardiac myosin essential light chain (*MYL3*), cardiac troponin I (*TNNI3*), cardiac troponin T2 (*TNNT2*), and tropomyosin 1 (*TPM1*). In addition, the genetic testing included sequencing the genes for alpha-galactosidase A (*GLA*), lysosomal associated membrane protein 2 (*LAMP2*), and AMP-activated protein kinase, gamma-2 subunit (*PRKAG2*), which are involved in storage diseases that can lead to cardiac hypertrophy [3]. The sequencing effort revealed two variants in the *MYH7 *gene: the proband was doubly heterozygous for a R719Q variant (2156G>A in Exon19) as well as a T1513S variant (4537A>T in Exon 33). The R179Q variant alone was likely to be causative for HCM as it met the criteria for pathogenity based on segregation studies and frequency [2, 4–7]. However, the significance of the T1513S variant was unclear even though it was detected in one additional HCM patient tested by the reference lab (http://www.hpcgg.org/LMM/) and in one patient reported in the literature [2]. In both of these prior cases, the T1513S variant was found in combination with the R719Q variant. Given the rare but recurrent presyncopal/syncopal complaints and significant family history of SCD, the proband received an internal cardiac defibrillator (ICD). The proband's 15-year-old sister was not found to have any genetic variants. 

To determine the significance of the *MYH7* T1513S variant, and to determine whether the two variants are present *in cis* (on the same allele) or *in trans* (on different copies of the gene), we contacted the proband's natural birth mother. The natural mother (36 years old) was found to have developed significant hypertrophic cardiomyopathy (IVS dimension of 2.7 cm, with a resting LV outflow gradient of 40 mmHg) with debilitating heart failure symptoms. She had received an ICD and was awaiting septal myomectomy. The genetic test on the mother revealed that she too was a heterozygote for both the R719Q and T1513S variants.

## 3. Discussion

Here, we present a case of a young adoptee with significant family history of HCM and sudden cardiac death who was closely monitored from age 6 to 17. This close follow-up revealed a pattern of natural history of HCM which may be generalizable. Firstly, her ECG began to show subtle changes as early as age 15, when her echocardiogram and MRI were normal. Then, at age 17, her echocardiogram showed diastolic dysfunction in the setting of minimal hypertrophy. Interestingly, an MRI obtained just two weeks later showed focal thickening in the anterior wall not previously seen by echocardiogram. These observations suggest that the appearance of ECG changes and diastolic dysfunction predate gross hypertrophy on echocardiogram, and that cardiac MRI is a more sensitive modality to detect focal hypertrophy [8].

The proband and her mother were both found to have the R719Q and T1513S variants in the *MYH7* gene, indicating that the two variants are on the same copy of the gene. In the only prior report of this variant combination, an implantable cardioverter defibrillator (ICD) was placed partly because of significant family of sudden cardiac death [2]. The proband reported here also has a very significant history of sudden cardiac death and early mortality due to HCM. Therefore, we suggest that the combination of the R719Q and T1513S variants *in cis* may be a “malignant” variant that imparts a poor prognosis in terms of the disease progression and the SCD risk. In the previous studies, the R719Q mutation was associated with divergent clinical presentations and prognoses: from mild hypertrophy without incidence of sudden death in a family of Hispanic origin [4] to high incidence of sudden death in a Chinese family [6]. However, the *MYH7* gene's exon 33, where the T1513S variant is situated, was not sequenced in either study, so it is possible that the Chinese family with the malignant phenotype carried an additional mutation *in cis*, resembling the R719Q and T1513S variant combination reported here. 

This case is notable for the fact that the proband was an adoptee whose adoptive parents have maintained contact with the natural maternal grandmother. This was critical in providing the ominous family history that compelled close cardiac follow despite the lack of overt evidence of HCM for many years. Finally, genetic testing of the affected natural mother provided the critical information establishing that the two variants were on the same copy of the *MYH7* gene. Thus, the proband has a 50% of chance of transmitting the disease genotype to each of her children rather than a 100% chance had the two variants been on separate chromosomes. This case highlights the importance of the need for close follow-up and comprehensive genetic evaluation of first degree relatives of patients with hypertrophic cardiomyopathy.
